# Neutrophilia with subclinical Cushing’s disease: A case report and literature review

**DOI:** 10.1515/biol-2022-0540

**Published:** 2023-01-24

**Authors:** Yan Zhang, Xiaoxi Lin, Fei Liu, Songtao Shou, Heng Jin

**Affiliations:** Department of Emergency Medicine, Tianjin Medical University General Hospital, Tianjin 300052, P.R. China

**Keywords:** subclinical Cushing's disease, silent corticotropin adenomas, leukocytosis, pituitary adenoma, case report

## Abstract

The increase in the level of neutrophils following subclinical Cushing’s disease is an uncommon clinical phenomenon that is characterized by insignificant biochemical or clinical evidence of hypercortisolism. In this study, we reported a 37-year-old female patient who presented with palpitations and fatigue, and showed increased neutrophils that were unaffected by anti-infection therapy. The patient was suspected of having a urinary tract infection because of occasionally with urinary frequency, urgency, increased procalcitonin, leukocytosis, and an increased proportion of neutrophils. The ineffectiveness of anti-infection therapy ruled out the possibility of urinary tract infection. Further examination of the bone marrow excluded the possibility of blood disease. However, the levels of blood cortisol and adrenocorticotropic hormone (ACTH) increased, and a magnetic resonance imaging examination revealed lesions in the sphenoidal sinus and sella area of the sphenoidal bone, which confirmed the relationship between increased glucocorticoids and increased neutrophils. This was further confirmed by follow-up surgery and pathological examination, which revealed silent corticotropin adenomas, which are characterized by the lack of biochemical or clinical evidence of hypercortisolism with positive immunostaining for ACTH.

## 1 Case presentation

A 37-year-old female patient was admitted to the Emergency Department of Tianjin Medical University’s General Hospital due to complaints of palpitations and fatigue for 1 week, which worsened over the last 4 days. One week before admission, the patient started having palpitations and fatigue, and occasionally with urinary frequency, without dysuria, fever, headache, dizziness, cough, abdominal pain, or diarrhea. She underwent a blood routine examination in another hospital, and the result showed an increase in the white blood cell count and the neutrophil percentage, a slight increase in procalcitonin (PCT), and five white blood cells/high power in urine. After 3 days of treatment with cephalosporin, the symptoms of palpitations and fatigue did not improve.

Since the onset of this illness, the patient had low energy levels, average appetite, good sleep, normal bowel movements, normal urination, and insignificant weight loss. There was no history of hypertension, diabetes, or coronary heart disease. The patient denied any history of hepatitis, tuberculosis, other infectious diseases, or any food allergies.

The patient presented at our hospital for further diagnosis and treatment. The white blood cell count and the percentage of neutrophils were high. The illness was still considered to be an infection, and the patient was treated with oral levofloxacin hydrochloride tablets. After administering the tablets for 3 days, the white blood cell count increased from 13.18 × 10^9^/L to 19.0 × 10^9^/L, and there was no change in the symptoms of palpitations or fatigue. The patient was admitted for further treatment.

At the time of admission, the patient’s body temperature was 36.2°C, pulse rate was 68 bpm, breathing rate was 17 bpm, and blood pressure was 120/72 mmHg. The patient showed mental anxiety, answered questions correctly, had a soft neck, no resistance, no thyroid enlargement, clear breath sounds in both lungs, no rales, good heart sounds, regular heart rhythms, a soft abdomen, no tenderness, rebound pain, and muscle tension. She had no percussion pain in the liver area, negative Murphy sign, no pain in the kidney area, bowel sounds four times per min, normal muscle strength in the limbs, and no swelling of the lower limbs.


**Informed consent:** Informed consent has been obtained from all individuals included in this study.
**Ethical approval:** The research related to human use has been complied with all the relevant national regulations, institutional policies and in accordance with the tenets of the Helsinki Declaration, and has been approved by the Medical Ethics Committee of “Tianjin Medical University General Hospital.”

## 2 Diagnosis and treatment

After admission, the serum levels of PCT, C-reactive protein (CRP), and interleukin-6 (IL-6) of the patient were in the normal range, blood (1–3)-β-D glucan and galactomannan (GM) tests were negative, and the temperature was normal. The results of laboratory tests are presented in Table 1.

**Table 1:** Results of laboratory tests

Variable	1 week before current admission	On admission	1 week after current admission	Reference range, adult
White cell count (×10^9^/L)	19.46↑	15.11↑	15.4↑	3.50–9.50
Hemoglobin (g/dL)	150	138	148	130–175
Platelets (×10^9^/L)	311	261	307	125–350
Neutrophils (%)	83.6↑	79.8↑	80.2↑	40–75
Neutrophil count (×10^9^/L)	16.28↑	12.05↑	12.35↑	1.8–6.3
Lymphocytes (%)	10.7	15	13.5	20–50
Lymphocyte count (×10^9^/L)	2.08	2.27	2.35	1.10–3.20
Monocytes (%)	5.2	4.4	5	3.0–10.0
Monocyte count (×10^9^/L)	1.01↑	0.67↑	0.77↑	0.10–0.60
Albumin (g/L)		32	37	35–55
Alanine aminotransferase (U/L)		37	68↑	5–40
Aspartate aminotransferase (U/L)		16	25	8–40
γ-glutamyl transferase (U/L)		18	27	7–49
Lactate dehydrogenase (U/L)		195	216	94–250
Urea nitrogen (mmol/L)		4.4	4.5	1.7–8.3
Serum creatinine (mmol/L)		47	47	44–115
Calcium (mmol/L)		2.06	2.33	2.15–2.55
Potassium (mmol/L)		3.5	4	3.5–5.3
Sodium (mmol/L)		143	138	136–145
Chloride (mmol/L)		107	101	96–108
FT3 (pmol/L)		3.55		2.43–6.01
FT4 (pmol/L)		11.13		9.01–19.05
T3 (nmol/L)		1.04		0.98–2.33
T4 (nmol/L)		86.54		62.68–150.84
Thyroid-stimulating hormone (µIU/mL)		1.191		0.350–4.940
Follicle-stimulating hormone (IU/L)			5.61	Follicle phase 3.03–8.08
Luteinizing hormone (IU/L)			2.22	Follicle phase 1.80–11.78
Prolactin (ng/mL)			6.62	Follicle phase 5.18–26.53
Cortisol (µg/dL)		34.3↑		5.00–25.00
ACTH (pg/mL)		73.3↑		0.00–46.00
Growth hormone (ng/mL)			<0.05	0.06–5.00
Erythrocyte sedimentation rate (ESR) (mm/h)		14		0–20
CRP (mg/L)		0.35		0.00–10.00
IL-6 (pg/mL)		<1.5		0.00–10.00
PCT (ng/mL)		<0.04		0.04–0.50
Serum amyloid A (mg/L)		14.05↑		<10.00

**Figure 1:**
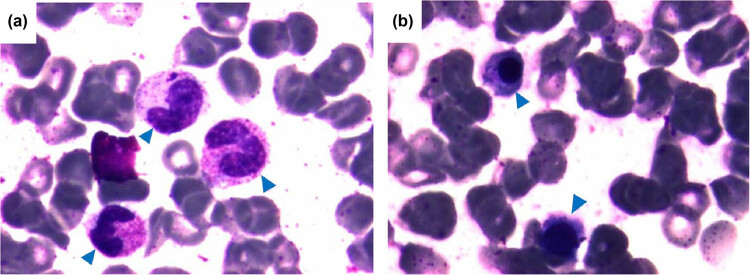
Bone marrow smear of the patient. Wright stain performs to visualize normal and mature granulocytes. (a) Neutrophils in the bone marrow (arrows). (b) Lymphocytes in the bone marrow (arrows).

We concluded that the increase in the neutrophil count was not related to infection. The bone marrow was aspirated to detect blood system diseases. Bone marrow hematopoiesis and the ratio of granule lines were normal, and there were no immature granulocytes (Figure 1), which excluded the possibility of hematological diseases.

**Figure 2:**
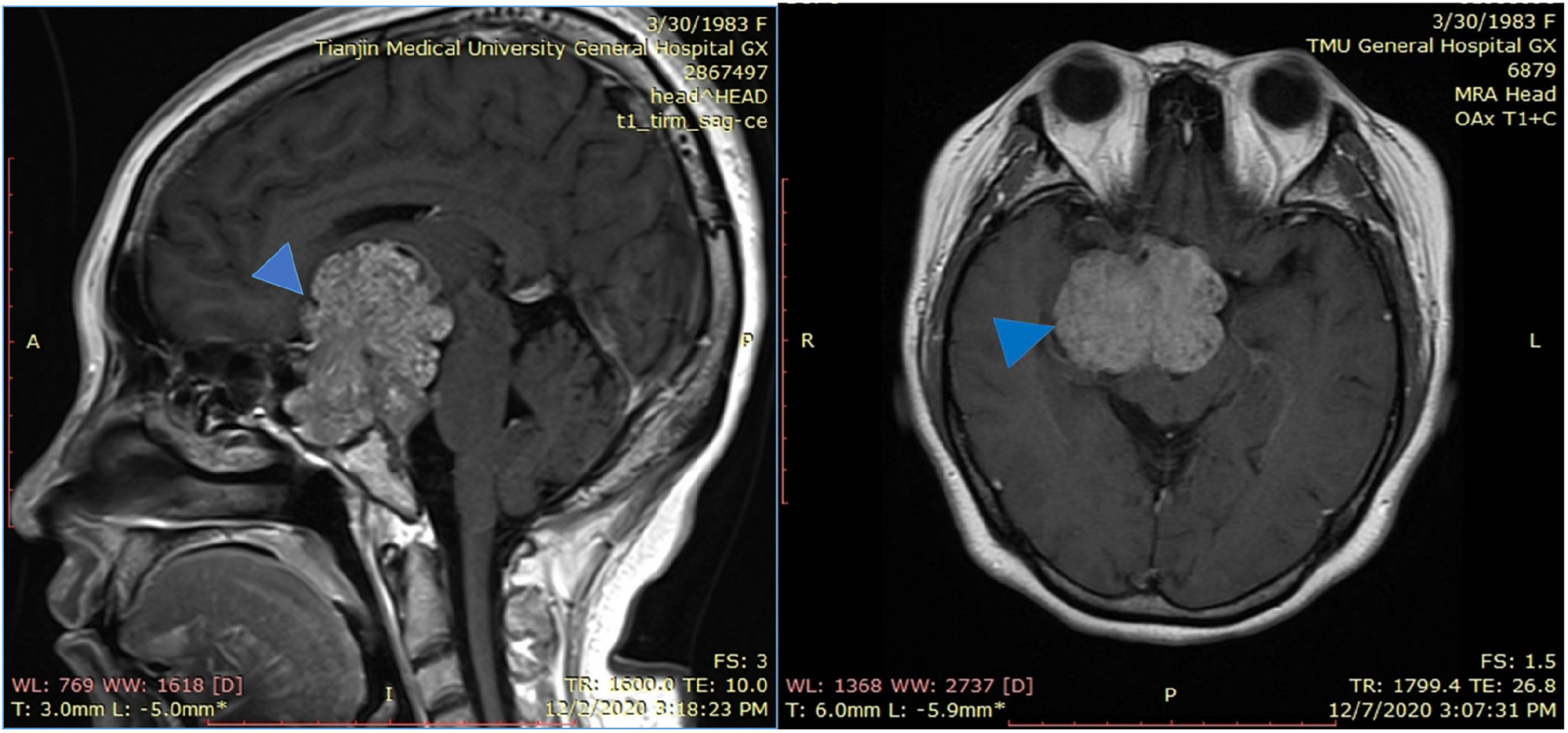
Enhancement of the pituitary gland determined via MRI. The tumor was located in the sella turcica and suprasellar region (arrows).

We also analyzed the serum level of granulocyte colony-stimulating factor (G-CSF), and the result was negative. However, blood adrenocorticotropic hormone (ACTH) and cortisol levels were elevated. We further examined the function of the adrenal cortex and found no abnormalities in the adrenal glands using ultrasound, while contrast-enhanced magnetic resonance imaging (MRI) examinations of the pituitary showed masses in the sella turcica and suprasellar regions, suggesting the presence of tumorous lesions of pituitary origin (Figure 2).

**Figure 3:**
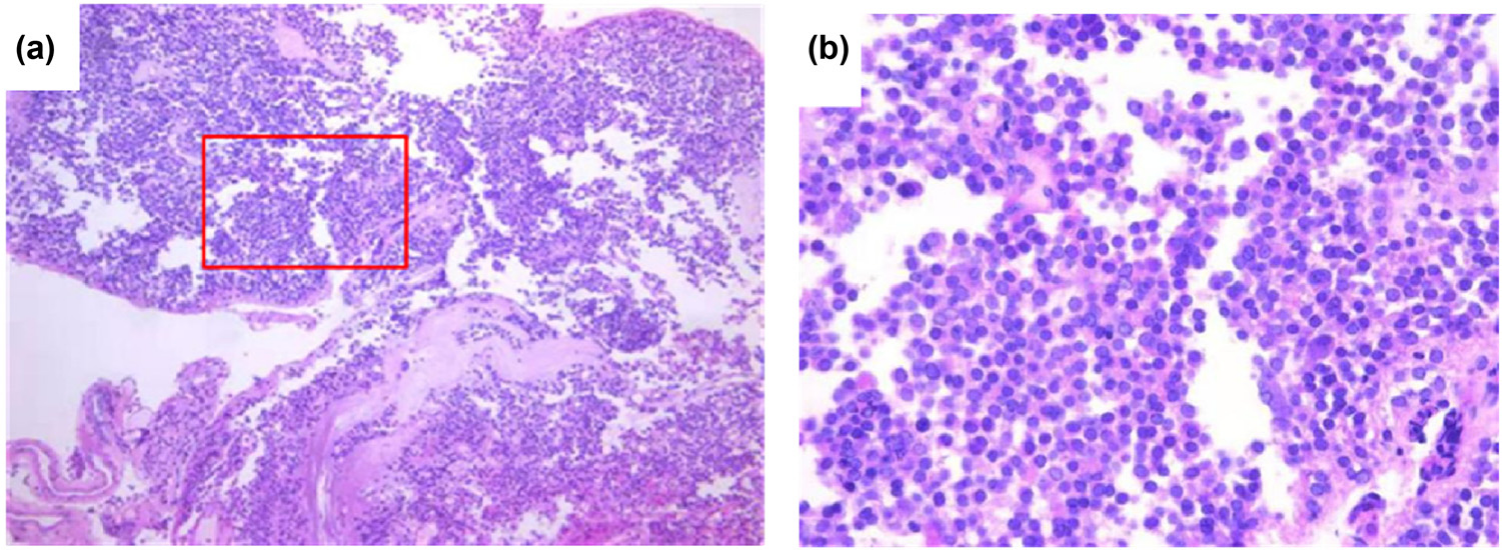
Pathological H&E staining of a pituitary tumor section (a: 100× and b: 400×). In panel (a), box with letter (b) was inserted and displayed the regions selected and enlarged at higher magnification.

Rough visual acuity in the ophthalmological consultation: 0.6 in the left eye, 0.1 in the right eye, and impaired rough visual field on both sides (left hemianopia). She underwent neurosurgery, and the pathological report indicated pituitary quiescent corticotropin cell adenoma (Figure 3). In the postoperative recheck, the blood parameters and adrenal corticosteroids were normal (including blood routine and glucocorticoid trend chart).

## 3 Pathological diagnosis

The patient was diagnosed with pituitary silent corticotropin adenomas (SCAs).

## 4 Discussion and review

Before admission, the patient occasionally showed frequent urination and urgency. The PCT, white blood cell count, and percentage of neutrophils increased. Urinary tract infection, which is also one of the causes of palpitations and fatigue, could not be ruled out. However, after the patient was admitted to the hospital, the PCT, CRP, and ESR were normal, and tests for viruses, fungi, and atypical bacteria were negative. Only neutrophils were elevated, which could not be decreased even by anti-infective treatment. In such cases, the causes of granulocyte increase other than infection should be considered. Therefore, bone marrow aspiration was performed. However, the results showed that bone marrow hematopoiesis was normal, and no immature granulocytes were found, ruling out the possibility of hematological diseases. Moreover, the patient was checked for rheumatism, tumor markers, thyroid function, blood G-CSF, and chest and abdomen CT. The results of these tests were negative, and the possibility of an increase in granulocytes caused by inflammatory factors to stimulate bone marrow hematopoiesis and abnormal secretion of CSF from the tumor was excluded. However, the levels of blood cortisol and ACTH were high, and further examination of the adrenal glands and pituitary gland showed that the pituitary space had lesions in the sphenoidal sinus and sellar area. Subsequently, the patient was transferred to the Department of Neurology for surgical resection, and the pathology was confirmed to be a pituitary tumor. Postoperative blood tests indicated that the levels of white blood cells and neutrophils, as well as blood cortisol concentration, returned to normal (Table 2). This further confirmed the correlation between the abnormal secretion of glucocorticoids and the increase in granulocytes.

**Table 2:** Comparison of blood tests

	On admission	Discharge	Reference range, adult
White cell count (×10^9^/L)	19.46↑	7.6	3.50–9.50
Neutrophils count (×10^9^/L)	16.28↑	4.56	1.8–6.3
Cortisol (µg/dL)	34.3↑	10.5	5.00–25.00
ACTH (pg/mL)	73.3↑	9.62	0.00–46.00

Clinically, the main cause of the increase in neutrophils could be divided into infectious and non-infectious diseases (Table 3). The site of infection might be systemic (sepsis) or focal and insidious, such as the lungs, urinary tract, intra-abdominal cavity, bone marrow, and endocardium.

**Table 3:** Differential diagnosis for neutrophilia [1,2,3]

**Non-infectious causes**	
Injury	Acute bleeding, myocardial infarction, pulmonary infarction/pulmonary embolism, and mesenteric rupture
Drugs	Vasoconstrictor drugs, glucocorticoids, heparin, lithium, G-CSF, and GM-CSF
Chronic inflammatory diseases	Rheumatic fever, connective tissue diseases such as systemic lupus erythematosus, myositis, and adult still disease
Solid tumors	Lung cancer, which may be related to the secretion of G-CSF by the tumor
Primary blood system diseases	Accumulation and diffusion diseases (e.g., polycythemia vera), abnormal proliferation syndrome, leukemia, chronic hemolysis, and the absence of spleen
Excessive synthesis of adrenal cortex hormones or thyroxine	Gradual increase in the number of white blood cells
Congenital diseases	Down syndrome and mutation of the CSF3R gene
**Infectious causes**	Bacteria, fungi, certain viruses, rickettsia, and other pathogenic microorganisms

From a pathophysiological perspective, the main reason for the increase in neutrophils in blood circulation is the increase in hematopoiesis or reserve release and the entry of cells from the marginated pool of blood vessels into the circulating pool [2,4,5]. Bacterial endotoxin stimulates hematopoiesis, which increases the number and proportion of granulocytes in the circulating pool. G-CSF is the main regulator that affects the production of physiological bone marrow granulocytes [6,7], including the induction of the differentiation of progenitor cells into the myeloid lineage, promotion of the proliferation of granulocyte precursor cells, reduction of the time of transition of the granulocyte compartment, and increase in the release of mature granulocytes. Hormones also change the number and proportion of granulocytes in the marginal pool and the circulating pool [8]. For example, glucocorticoids can increase the number of granulocytes in the marginal pool and the circulating pool, while epinephrine only promotes the entry of granulocytes from the marginal pool to the circulating pool [9].

Cushing’s syndrome, also known as hypercortisolism, was first reported by Harvey Cushing in 1912 [10]. It is mostly diagnosed based on the patient’s medical history, clinical manifestations, and auxiliary test results. Patients in the subclinical stage may present weight gain, fatigue, weakness, delayed wound healing, easy bruising, back pain, bone pain, mental and emotional issues, loss of libido, erectile dysfunction in males, irregular menstrual cycles in females, infertility, hyperhidrosis, hirsutism, and biparietal visual loss if there are large pituitary adenomas, recurrent fungal, and bacterial infections due to impaired immunity, and difficulty in combing hair or rising from a sitting position [11]. Psychological problems, such as cognitive dysfunction and depression, are common [12]. Some patients may develop severe osteopenia and bone fractures. The most common symptoms include abdominal obesity but with thin arms and legs, reddish stretch marks, a round red face, a lump of fat between the shoulders, weak muscles, weak bones, acne, and fragile skin that heals poorly. The less common symptoms include periods of mental changes with depression, anxiety, moodiness, or differential behavior, severe fatigue, headaches, thirst, and increased urge to urinate, as well as high blood pressure, blood sugar, cholesterol, and triglycerides [13,14]. The main diagnostic tests include 24-h urine-free cortisol, the dexamethasone suppression test, salivary and blood serum cortisol tests, examination of the ACTH levels, and rarely, an ACTH stimulation test [15,16]. Inferior petrosal sinus sampling (IPSS) or bilateral IPSS (BIPSS) is a more accurate test to differentiate pituitary from ectopic or adrenal Cushing’s syndrome [15], which is the gold standard to distinguish between Cushing’s disease and ectopic hypercortisolism [17].

Diagnosis based on etiology and localization is also essential. Intrinsic conditions include a clinical syndrome characterized by hypercortisolemia caused by various etiologies. Performing abdominal CT or pituitary MRI examinations might be the fastest and most accurate way to detect tumors. However, only 48% of the pituitary tumors are detected by performing MRI before surgery [18]. Tumors identified via MRI and confirmed by surgery have an average size of 6 mm [18]. The common cause of such tumors is the excessive secretion of ACTH by the pituitary, followed by adrenal cortex tumors and ectopic ACTH syndrome. Cushing’s disease is most often caused by a pituitary adenoma (specifically pituitary basophilism) or excess production of the corticotropin-releasing hormone that stimulates the synthesis of cortisol by the adrenal glands. Pituitary adenomas account for 80% of the cases with endogenous Cushing’s disease [19]. Long-term use of higher doses of glucocorticoids or ACTH can cause clinical manifestations such as Cushing’s syndrome, which is then called iatrogenic Cushing’s syndrome. Excess alcohol consumption can also cause similar problems [11]. The first line of treatment for hypercortisolism due to Cushing’s disease, adrenal tumor, or ectopic tumor is surgical resection [20]. However, sometimes, especially in Cushing’s disease, the tumors lack a distinct border with the pituitary gland, which greatly increases the risk of either recurrence or pituitary insufficiency after surgical resection [21]. Reoperation after initial surgery is necessary as long as the disease persists [20]. Pituitary radiation therapy is recommended for the treatment of postoperative persistent hypercortisolemia following unsuccessful transsphenoidal surgery or Cushing’s disease with mass effect or invasion of surrounding structures. Bilateral adrenalectomy is another strategy to immediately reduce the level of cortisol and control hypercortisolism [22]. This surgical operation may cause permanent adrenal cortical dysfunction, which needs corticosteroid replacement therapy throughout life [23]. The common complication that occurs after this surgery is Nelson’s syndrome, which is characterized by progressive cutaneous melanosis and enlargement of pituitary adenoma [24]. Another treatment strategy is the administration of oral medication. Inhibitors of steroid synthesis can inhibit cortisol synthesis but do not have a direct therapeutic effect on tumors or restore the normal function of the hypothalamic–pituitary–adrenal axis [25]. The commonly used drugs in this category include mitotane, amino-glutamate, ketoconazole, and etomidate [26,27]. Mifepristone, a glucocorticoid receptor antagonist, can relieve clinical symptoms but has negligible effects on pituitary and adrenal diseases [28]. It is suitable for patients who cannot undergo surgery. Strict monitoring is required during medication [29]. In addition to the management of Cushing’s syndrome, the treatment of comorbidities, such as diabetes mellitus, bone fractures due to osteoporosis, psychiatric issues, high blood pressure, kidney stones, peptic ulcer disease, electrolyte disorders, and susceptibility to infections, is also crucial [30,31]. The morbidity, mortality, and prognosis of Cushing’s syndrome are primarily related to complications and comorbidities [32].

Subclinical Cushing’s syndrome is a condition of hypercortisolism that occurs without typical signs and symptoms. It is associated with an adrenal mass and an increased risk of metabolic diseases, such as diabetes, hypertension, fragile fractures, cardiovascular events, and mortality [33]. Subclinical Cushing’s syndrome is not rare and is estimated to affect 0.2–2% of the adult population [34,35]. However, subclinical Cushing’s disease is defined as ACTH-induced mild hypercortisolism without the typical clinical symptoms of Cushing’s disease [36]. The first case of subclinical Cushing’s disease was reported by Kageyama et al. [37]. The diagnostic criteria were established in Japan in 2010, which included the presence of a pituitary adenoma on MRI, normal-to-high plasma ACTH levels, and normal cortisol levels in the morning, without a typical Cushingoid appearance [38]. Secretion of the inactive ACTH precursor and glucocorticoid resistance at the transcriptional level are two molecular pathogeneses of subclinical Cushing’s disease that might vary from classical Cushing’s disease [39]. SCAs are non-functional pituitary adenomas with positive immunostaining for ACTH. Unlike secretory ACTH pituitary adenomas in patients with Cushing’s disease, SCAs do not show biochemical or clinical signs of hypercortisolism. Instead, most SCAs are macroadenomas and are clinically non-functional adenomas (NFAs) that require resection due to mass-occupying effects [36]. Few SCAs develop into overt Cushing’s disease. The treatment of SCAs is the same as that for normal pituitary tumors. However, the recurrence rate of SCA tumors after surgery is high, which makes regular follow-up examinations essential [40].

Masri-Iraqi et al. [41] investigated the prevalence of leukocytosis in patients with Cushing’s disease and reported that patients with Cushing’s disease present with leukocytosis in approximately 40% of the cases. However, patients with subclinical Cushing’s disease due to pathological confirmed SCAs presented with elevated neutrophils are rare. This case indicated that leukocytosis might occur in subclinical Cushing’s disease.
